# Intra-articular injections of sodium hyaluronate (Hyalgan^®^) in osteoarthritis of the knee. a randomized, controlled, double-blind, multicenter trial in the asian population

**DOI:** 10.1186/1471-2474-12-221

**Published:** 2011-10-06

**Authors:** Teng-Le Huang, Chi-Ching Chang, Chian-Her Lee, Shih-Ching Chen, Chien-Hung Lai, Ching-Lin Tsai

**Affiliations:** 1 Department of Orthopaedic, China Medical University Hospital, No.2 Yu-Der Road, Taichung 404, Taiwan; 2 Department of Sports Medicine, College of Health Care, China Medical University, No.91, Hsueh-Shih Road, Taichung, Taiwan 404, Taiwan; 3 School of Medicine, College of Medicine, China Medial University, No.91, Hsueh-Shih Road, Taichung, Taiwan 404, Taiwan; 4 Department of Medicine, Division of Rheumatology, Immunology, Allergy, Taipei Medical University Hospital, 250 Wu-Hsing Street, Taipei City 110, Taiwan; 5 Department of Orthopaedic, Taipei Medical University Hospital, 250 Wu-Hsing Street, Taipei City 110, Taiwan; 6 Department of Physical Medicine and Rehabilitation, Taipei Medical University Hospital, 250 Wu-Hsing Street, Taipei City 110, Taiwan; 7 Department of Physical Medicine and Rehabilitation, Taipei Medical University Hospital, 250 Wu-Hsing Street, Taipei City 110, Taiwan; 8 Department of Orthopaedic, National Taiwan University and University Hospital, 7 Chung Shan South Road, Taipei, Taiwan

## Abstract

**Background:**

The efficacy and tolerability of 500-730 kDa sodium hyaluronate (Hyalgan^®^) for treatment of osteoarthritis (OA) pain has been established in clinical trials, but few data are available in the Asian population. We conducted a randomized, double-blind, multicenter, placebo-controlled study to evaluate the efficacy and tolerability of this preparation in a Taiwanese population.

**Methods:**

Two hundred patients with mild to moderate OA of the knee were randomized to receive five weekly intra-articular injections of sodium hyaluronate or placebo. The primary efficacy outcome was the change from baseline to Week 25 in patients' evaluation of pain using a 100-mm visual analog scale (VAS) during the 50-foot walking test. Additional outcomes included Western Ontario and McMaster Universities (WOMAC) scores, time on the 50-foot walking test, patient's and investigator's subjective assessment of effectiveness, acetaminophen consumption, and the amounts of synovial fluid.

**Results:**

The Hyalgan^® ^treatment group showed a significantly greater improvement from baseline to Week 25 in VAS pain on the 50-foot walking test than the placebo group (p = 0.0020). The Hyalgan^® ^group revealed significant improvements from baseline to week 25 in WOMAC pain and function score than the placebo group (p = 0.005 and 0.0038, respectively) Other outcomes, such as time on the 50-foot walking test and subjective assessment of effectiveness, did not show any significant difference between groups. Both groups were safe and well tolerated.

**Conclusions:**

The present study suggests that five weekly intra-articular injections of sodium hyaluronate are well tolerated, can provide sustained relief of pain, and can improve function in Asian patients with osteoarthritis of the knee.

**Level of Evidence:**

Therapeutic study, Level I-1a (randomized controlled trial with a significant difference).

**Trial registration:**

ClinicalTrials.gov Identifier: NCT01319461

## Background

Osteoarthritis (OA) of the knee is a common arthropathy and a leading cause of disability in elderly adults. The disease is characterized by a reduction in the lubricating and viscoelastic properties of the synovial fluid, accompanied by progressive destruction of the cartilage surface [1,2]. When simple analgesics, such as acetaminophen, prove ineffective for reducing the pain of OA, the most common therapy for treating the signs and symptoms of OA is nonsteroidal anti-inflammatory drugs (NSAIDs). NSAIDs have proven efficacy for the relief of pain and inflammation of OA, but their use may be restricted by adverse gastrointestinal effects, including serious occurrences of bleeding [3,4].

Hyaluronic acid present in the synovial fluid and cartilage of the knee imparts viscoelastic properties that allow for the efficient movement of articular joints [5]. Hyalgan^® ^(Fidia, S.p.A., Abano Terme, Italy) is a sodium hyaluronate preparation, with a molecular weight of 500-730 kDa, which is naturally derived from rooster combs and has the same molecular structure as the endogenous hyaluronic acid present in the human body [6]. This sodium hyaluronate restores the viscoelastic properties of the synovial fluid and stimulates synthesis of endogenous hyaluronic acid by synoviocytes [7,8]. In addition, this sodium hyaluronate may also mediate therapeutic effects in OA by a number of other biochemical actions within the joint, including the induction of proteoglycan aggregation and proteoglycan synthesis, inhibition of inflammatory mediators, and analgesic activity [9].

The efficacy and tolerability of intra-articular (i.a.) injections of this sodium hyaluronate have been established in many clinical trials conducted worldwide [10-18]. In a large, controlled clinical trial conducted in the United States, a 5-injection course of sodium hyaluronate was shown to provide effective pain relief for as long as 26 weeks after initiation of the therapeutic course [10]. Accordingly, i.a. injections of sodium hyaluronate are considered by the American College of Rheumatology (ACR) to be an alternative to NSAIDs for treatment of pain in patients with OA [4]. Hyalgan^® ^was approved by the United States Food and Drug Administration in 1997, and this therapeutic agent is now available in over 43 countries worldwide. However, only one study has been published evaluating this sodium hyaluronate in an Asian population; these investigators found that 4 weekly doses decreased pain and increased mobility over a 49-day follow-up [19]. The primary purpose of this study was to evaluate the efficacy and tolerability of this 20-mg/2 mL sodium hyaluronate administered as 5 i.a. injections at weekly intervals, for relieving the pain of OA of the knee in the Asian population.

## Methods

### Patients

Patients eligible for the study were males or females > 50 years of age, diagnosed with OA of the knee according to ACR criteria (knee pain with one or more of the following conditions: age > 50 years, crepitus, or morning stiffness < 30 minutes in duration). Eligible patients also had radiographic evidence of OA with Kellgren-Lawrence score of II to III (mild to moderate) on x-ray, with predominance in the tibio-femoral compartment and visual analog scale (VAS) pain scores of ≥ 40 mm on a 50-foot walking test. It was required that any acute disease or trauma leading to secondary OA must have occurred at least 5 years before study entry. Major exclusion criteria included severe degeneration of the knee joint with marked joint narrowing, varus, or valgus deformity of the knee (> 12°) or other joint deformities, or other joint disorders (eg, inflammatory joint disease, specific arthropathy, severe axis deviations or instabilities, joint or skin infections, joint prostheses of the lower limbs or symptomatic hip). Patients were not permitted to have received i.a. steroid injections within the 2 weeks prior to study entry.

### Study design

Ethical approval has been granted by Ethics Committee of Department of Health, Taiwan (Ref: DOH-TW-0890036458) and the participating medical centers, including National Taiwan University Hospital, the Taipei Medical College Hospital, and the Tri-Service General Hospital, in Taipei, Taiwan.

This was a prospective, randomized, masked-observer, double-blind, parallel group, placebo-controlled study designed to evaluate the efficacy and safety of 5 weekly i.a. injections of sodium hyaluronate (Hyalgan^®^, Fidia S.p.A, Abano Terme, Italy) at 20 mg/2 mL in comparison with 5 weekly i.a. injections of saline placebo (2 mL) in patients with radiographically confirmed, mild to moderate OA of the knee. The study was conducted at 3 hospitals: the National Taiwan University Hospital, the Taipei Medical College Hospital, and the Tri-Service General Hospital, in Taipei, Taiwan. Six investigators enrolled patients. Efficacy was defined as improvement from baseline to Week 25 after initiation of treatment in 100-mmVAS pain on the 50-foot walking test. After a screening period, eligible patients were randomized to 5 weekly injections with either sodium hyaluronate or placebo, and were then evaluated at Week 5, Week 13, and Week 25. Patients who required further pain treatment were permitted to take acetaminophen as needed, but not exceeding 3 g/day. Patients were not permitted to take any acetaminophen on the day before the study visit. Oral and parenteral corticosteroids, i.a. corticosteroid injections, NSAIDs or analgesics other than acetaminophen, topical analgesic preparations, rehabilitation, physical therapy, or acupuncture were not permitted during the study.

### Definition of the primary outcome and the secondary outcome

The primary efficacy outcome was defined as the change from baseline (Week 0) to Week 25 in patients' evaluation of pain, measured by a 100-mm VAS during the 50-foot walking test (0 = "No Pain" and 100 = "Maximum Pain"). The secondary efficacy measures included: (1) the Western Ontario and McMaster Universities (WOMAC) OA scales for pain, stiffness, and physical function, by VAS; (2) time taken in the 50-foot walking test; (3) volume of synovial effusion of the enrolled knee, if present; (4) overall effectiveness evaluated by patients and investigators on a scale of 1 to 6 (1 = Gravely worsened and 6 = Excellent improved); and 5) acetaminophen tablet count.

### Sample Size

The sample size required was estimated based on results from the UK study conducted by Huskisson and Donnelly, in which the adjusted means difference between treatment groups for VAS pain on movement at 6 months was equal to 15.04 mm, and the corresponding effect size was equal to 0.52 for completers [18]. Sixty patients in each group were required to ensure adequate power to detect a similar between-groups difference.

### Statistical Analysis

Treatment groups were compared with respect to the primary efficacy variable using an analysis of variance, including treatment, investigator, and treatment-by-investigator interaction as factors. The analysis was based on the intent-to-treat (ITT) population with the last observation carried forward (LOCF) for patients who did not complete the study. Improvement in WOMAC index and improvement in time taken for the 50-foot walking test were analyzed using the same approach as for the primary variable. Volume of synovial fluid effusion was evaluated using the student's t-test. Patients' and investigators' overall effectiveness evaluations were analyzed by Chi-square test. Between-group comparison for acetaminophen consumption was conducted using the Wilcoxon rank sum test.

### Safety

Safety was assessed by adverse events, laboratory findings, and vital signs.

## Results

### Patient Characteristics and Disposition

**Figure 1:**
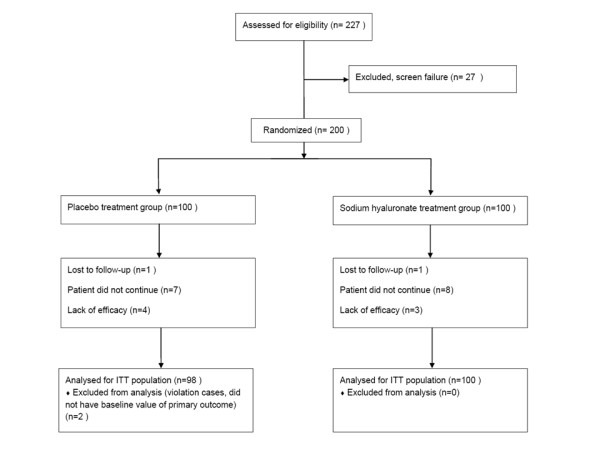
**Flow of study participants**.

Of 227 patients screened, 200 patients were randomized in the study. Of these, 176 patients completed the study, and 198 patients (100 in the sodium hyaluronate group, 98 in the placebo group) were included in the ITT population. Of the 24 patients who withdrew from the study, 2 were lost to follow-up, 7 withdrew due to lack of efficacy, and 15 withdrew consent. The flow of study participants is shown in Figure 1.

Demographic and baseline characteristics of the study population are shown in Table 1. Mean age of the patients in both treatment groups was 65.0 years. The two treatment groups were similar with regard to all demographic and baseline disease characteristics evaluated. There were no significant differences between the treatment groups with regard to baseline VAS scores on the 50-foot walking test or any of the other measures of pain and function.

**Table 1:** Baseline demographics and disease characteristics of all randomized patients

	Total (n = 200)	Hyalgan^® ^(n = 100)	Placebo (n = 100)	*p *value
**Demographic Characteristic**				
Age, mean (SD), yrs	65.0 (8.3)	65.9 (8.1)	64.2 (8.4)	NS
Sex, n (%)				
Male	48 (24.0%)	26 (26.0%)	22 (22.0%)	NS
Female	152 (76.0%)	74 (74.0%)	78 (78.0%)	NS
Weight, mean (SD), kg	62.7 (10.2)	63.3 (11.9)	62.1 (8.3)	NS
Height, mean (SD), cm	156.5 (7.2)	156.8 (7.7)	156.3 (6.7)	NS
BMI, mean (SD), kg/m^2^	25.6 (3.6)	25.7 (4.3)	25.4 (2.9)	NS
**Disease Characteristic**				
Kellgren-Lawrence grade				
Grade II (mild), n (%)	119 (59.5%)	59 (59.0%)	60 (60.0%)	NS
Grade III (moderate), n (%)	81 (40.5%)	41 (41.0%)	40 (40.0%)	NS
Duration of OA, mean (SD), days	427.0 (1022.5)	499.2 (1190.8)	354.8 (820.5)	NS
**Clinical Evaluation**				
Pain on 50-foot walking test (VAS 0-100 mm) mean (SD)	46.75 (10.59)	47.85 (10.76)	45.65 (10.36)	NS
WOMAC A-pain (VAS 0-100 mm) mean (SD)	45.56 (12.12)	45.73 (11.17)	45.39 (13.06)	NS
WOMAC C-function(VAS 0-100 mm) mean (SD)	45.99 (12.23)	46.54 (11.31)	45.45 (13.13)	NS

SD, standard deviations.

NS, non-significantly different between treatment groups (P > 0.05)

### Primary Efficacy Outcome

**Figure 2:**
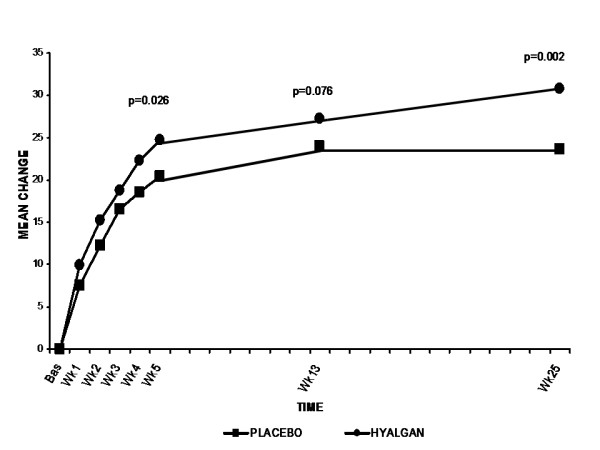
**Mean change from baseline in VAS pain on the 50-foot walking test**. The primary efficacy outcome of change from baseline through Week 25 measured by VAS pain score (0-100 mm scale) on the 50-foot walking test for the ITT population (sodium hyaluronate, n = 100; placebo, n = 98).

Concerning the placebo group, the VAS scale was improved from baseline (Week 0) of 45.15 mm to 21.53 mm (Week 25). As to Hyalgan treatment group, the VAS scale was improved from 47.85 (Week 0) to 17 mm (Week 25). The difference of VAS scale at week 25 between these two groups was statistically significant (17 mm versus 21.53 mm for Hyalgan and placebo, respectively) (p = 0.035). (Table 2) The Hyalgan treatment group showed a significantly greater improvement from baseline to Week 25 in VAS pain on the 50-foot walking test than the placebo group (reduction of 30.85 mm versus 23.62 mm for Hyalgan and placebo, respectively; and, least squares mean difference between groups of 8.07 ± 2.58 mm, 95% CI 2.98-13.16 mm, *p *= 0.002). (Table 2) The reduction in pain intensity as compared with baseline (week 0) was apparent at one week after the first dose (reduction of 9.9 ± 12.33 mm versus 7.55 ± 8.95 mm for sodium hyaluronate and placebo, respectively). However, the difference between groups became significant only after completion of the 5-injection treatment course (Week 5) (p = 0.026). (Figure 2) The detailed VAS scales at different period were shown in Table 2. The Mean changes of VAS scale from baseline (W0) in both groups were presented in Figure 2.

**Table 2:** The VAS pain scales of Placebo and Hyalgan treatment groups on the 50-foot walking test

Week (W)	Hyalgan^® ^(n = 100) VAS (mm)	Placebo (n = 98) VAS (mm)	*p *value
Baseline (W0)	47.85 ± 10.76	45.15 ± 9.75	0.066
W25	17.00 ± 14.32	21.53 ± 15.69	0.035*
Change from W0 to W1	9.9 ± 12.33	7.55 ± 8.95	0.127
Change from W0 to W5	24.75 ± 12.66	20.41 ± 15.38	0.026
Change from W0 to W13	27.27 ± 14.97	24.01 ± 16.95	0.076
Change from W0 to W25 (Primary Outcome)	30.85 ± 14.16	23.62 ± 16.38	0.002**

VAS pain scales were shows as mean ± SD.

*P < 0.05, **P < 0.01, indicated significantly different between treatment groups.

### Secondary Efficacy Outcomes

#### WOMAC scores

**Figure 3:**
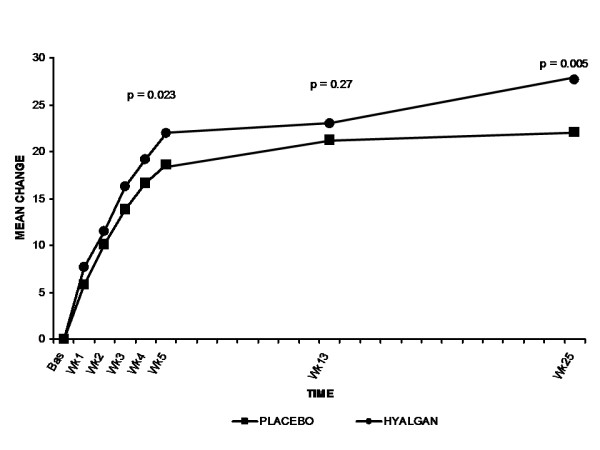
**Mean change from baseline (mm) in WOMAC pain scores**. Patients treated with sodium hyaluronate exhibited statistically significant improvement in WOMAC pain indices as compared with placebo treatment. Data are shown for the ITT population.

**Figure 4:**
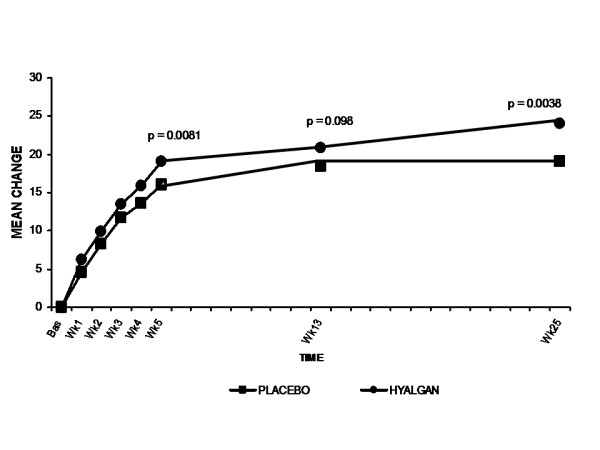
**Mean change from baseline (mm) in WOMAC function scores**. Patients treated with sodium hyaluronate exhibited statistically significant improvement in WOMAC function indices as compared with placebo treatment. Data are shown for the ITT population.

Both the sodium hyaluronate and placebo groups showed improvement in the secondary efficacy parameters of WOMAC pain, stiffness, and function scores over the 25-week study period. The sodium hyaluronate group revealed significant improvements from baseline to week 25 in WOMAC pain and function score than the placebo group (p = 0.0050 and 0.0038, respectively) Although there was a greater improvement in sodium hyaluronate-treated patients for the parameter of WOMAC stiffness throughout the 25-week follow-up period, this difference did not achieve statistical significance at any timepoint. (Table 3) The between-group mean difference for WOMAC pain scores was significant in favor of sodium hyaluronate as early as Week 5 (p = 0.023) (Figure 3). Similarly, patients treated with sodium hyaluronate showed significantly greater improvement in WOMAC function scores as early as the end of the 5-injection treatment course (Week 5) (p = 0.0081) (Figure 4).

**Table 3:** Mean change from baseline to Week 25 in WOMAC index scores

WOMAC section	**Hyalgan**^**® **^**(n = 100)**	Placebo (n = 98)	*p *value
**Pain**			
Change from W0 to W25, mm (SE)	29.28 (1.92)	21.52 (1.94)	0.0050*
**Stiffness**			
Change from W0 to W25, mm (SE)	24.85 (2.18)	22.58 (2.21)	0.4640
**Function**			
Change from W0 to W25, mm (SE)	25.16 (1.67)	18.20 (1.69)	0.0038*

SE, standard error of mean.

*P < 0.05, indicated significantly different between treatment groups.

#### Time on the 50-foot walking test

Both treatment groups showed a slight reduction in time consumed on the 50-foot walking test with no statistically significant difference between them.

#### Patient's and investigator's assessment of effectiveness

Nominally more patients in the sodium hyaluronate group compared with the placebo group were considered "slightly improved", "improved", or "excellent improved" by both patient's judgment of effectiveness at Week 25 (58 versus 45 patients, respectively) and investigator's judgment (60 versus 47 patients, respectively). On the other hand, less patients in the sodium hyaluronate group compared with the placebo group were considered "slightly worsened" or "gravely worsened" at Week 25 by both patient's judgment (6 versus 15 patients, respectively) and investigator's judgment (7 versus 14 patients, respectively). However, the differences did not achieve significance.

#### Acetaminophen consumption

There was no statistically significant difference between the treatment groups with regard to the total mean acetaminophen consumption over the 25-week study period.

#### Synovial fluid volume

The amounts of synovial fluid drawn at each timepoint through Week 25 were similar in these two groups, either for the entire treatment population or when the analysis was only applied to patients with the volume of synovial fluid > 0.

### Safety

There were a total of 153 adverse events (67 in the sodium hyaluronate groups, 86 in the placebo group) reported by 87 patients. More patients in the placebo group compared with the sodium hyaluronate group experienced at least 1 adverse event (48% versus 39%). The nature of adverse events reported was comparable between these two treatment groups. All reported events were mild or moderate in intensity, and none were considered related to study treatment. The most frequently reported adverse events in both treatment groups were in the respiratory system, the most common of which were acute bronchitis and bronchitis combined, occurring in a total of 10 patients in the study. No patients discontinued from the study because of an adverse event. Five serious adverse events were reported in the study: 3 in the sodium hyaluronate group and 2 in the placebo group. These included a forearm fracture, intestinal obstruction, and aggravated urinary incontinence in the sodium hyaluronate group, and upper gastrointestinal bleeding and joint sprain in the placebo group. All were considered to be unrelated to study treatment.

A statistically significant treatment difference was seen in change from baseline for platelet counts at Week 5 (p = 0.027), with standardized counts decreasing slightly in the sodium hyaluronate group and increasing slightly in the placebo group. However, this difference was not considered clinically relevant nor was it apparent at other timepoints during the study. No other significant laboratory results or vital signs findings were observed.

### Power of the Current Trial

Based on the current observed results of primary outcome (change from W0 to W25) and the sample size for each group, this study give a power of 91.3% to identify the change in VAS score between groups.

## Discussion

Hyaluronan (HA), a large glycosaminoglycan composed of repeating disaccharides of D-glucuronic acid and N-acetyl-glucosamine, is a ubiquitous component of the extracellular matrix. Intra-articular (i.a.) injection of HA is now applied worldwide for the treatment of arthritis. The efficacy and tolerability of i.a. injections of Hyalgan^® ^have been established in many clinical trials conducted worldwide [10-19]. However, little study has specially addressed in evaluating the effect and safety of this sodium hyaluronate on an Asian population.

The primary aim of this clinical trial was to study the efficacy and safety of 5 weekly i.a. injections of sodium hyaluronate (Hyalgan^®^), as compared with the placebo, in Asian patients suffering from OA of the knee. The duration of this study comprised a 5-week treatment period followed by a 20-week follow-up period. The results demonstrate that the Hyalgan treatment group showed a significantly greater improvement from baseline to Week 25 in VAS pain on the 50-foot walking test than the placebo group. Our findings were further supported by a previous pivotal clinical trial involving 218 completed patients (103 for Hyalgan^® ^versus 115 for placebo) [10]. At Week 26 of follow-up, the statistically significant treatment difference for pain intensity on the 50-foot walking test (assessed by a 0-100 mm VAS) was 8.8 mm in favor of sodium hyaluronate, similar to the magnitude of effect seen here (8.07 mm). Their sample size and the result of VAS difference are quite comparable to our study. That means the current study provide a similar power as theirs. As to the sample size and the accepted absolute changes of VAS, Huskisson [18] conducted a randomized control trial to examine the efficacy, safety and patient satisfaction of i.a.HA in patients with osteoarthritis of the knee. They used a sample size of 50 patients for each group to give a power of around 90% for the detection of a mean treatment change of 15.4 mm in VAS score. In the current study, our sample size is 100 patients for each group that provide a similar power of 91.3% for the detection of mean treatment change of 8.07 mm in VAS score.

In the present study, the reduction in pain intensity was apparent at one week after the first dose of sodium hyaluronate. This finding was further confirmed by some recent reports. Lee [20] conducted an open-label, randomized, multicentre clinical trial to compare the clinical effects between high and low molecular weight HA. They indicated that the symptom relief could be as soon as one week after HA injection, in terms of significantly reducing the VAS and improving the WOMAC pain, function, and stiffness scores. A similar finding was shown in another clinical trial by Kirchner M [21]. We may attribute the rapid reduction in pain intensity to the mechanism of anti-inflammation through the synovium, which was further supported by a recent review article [22].

Regarding the secondary outcome estimated by WOMAC scores, we found that the pain and function sub-scales were significantly reduced as early as at Week 5, by compared with placebo group. Furthermore, the significant difference was maintained throughout the study. As to the subjective evaluations, more patients in the sodium hyaluronate group compared with the placebo group were considered "slightly improved", "improved", or "excellent improved" by both patient's and investigator's judgment of effectiveness at Week 25. These results all imply that i.a. administration of Hyalgan^® ^can improve joint function and ameliorate pain in Asian patients with knee osteoarthritis.

Concerning the estimation of safety, both sodium hyaluronate and placebo were well tolerated in this study. There were no instances of any severe local inflammatory reactions, such as have been reported with growing frequency in the literature for another hyaluronate product, hylan G-F 20 [23]. Aside from an apparently isolated difference between the treatment groups in platelet counts at Week 5 (not clinically significant), no changes in laboratory values, blood pressure, pulse rate, or body temperature were observed in either treatment group.

In the present study, efficacy of sodium hyaluronate, as measured by the primary outcome variable, did not show signs of waning at the 25-week timepoint. Therefore, we might have seen a longer-term benefit of this sodium hyaluronate had the follow-up period been extended. This finding of a relatively long-term pain relief is also consistent with findings of controlled and uncontrolled clinical trials that found continuing benefits with sodium hyaluronate treatment for at least 6 months [18] and, in some cases, for 1 or more years after completion of the treatment course [16,24,25].

To sum up, our results showed that a 5-injection course of this sodium hyaluronate was effective, in terms of a significantly greater improvement from baseline to Week 25 in VAS score, WOMAC pain and function score than the placebo group. The whole course was safe and well tolerated both in sodium hyaluronate treatment group and the placebo.

## Conclusion

The present study demonstrated that intra-articular administration of Hyalgan^® ^is safe and can improve joint function and ameliorate pain in Asian patients with knee osteoarthritis.

## Competing interests

The authors declare that they have no competing interests.

## Authors' contributions

All authors have read and approved the final manuscript.

TL H drafted the manuscript, interpreted of data and participated in its design and coordination. CC C, SC C, and CH L investigated and followed patients. CH L investigated, followed, and clinically managed patients. CL T initiated the study and participated in its design and coordination.

## Pre-publication history

The pre-publication history for this paper can be accessed here:

http://www.biomedcentral.com/1471-2474/12/221/prepub
